# Crossed Cerebellar Diaschisis Indicates Hemodynamic Compromise in Ischemic Stroke Patients

**DOI:** 10.1007/s12975-020-00821-0

**Published:** 2020-06-06

**Authors:** Lita von Bieberstein, Christiaan Hendrik Bas van Niftrik, Martina Sebök, Mohamad El Amki, Marco Piccirelli, Christoph Stippich, Luca Regli, Andreas R. Luft, Jorn Fierstra, Susanne Wegener

**Affiliations:** 1 Dept. of Neurology, Clinical Neuroscience Center, University Hospital Zurich and University of Zurich, Frauenklinikstrasse 26, 8091 Zurich, Switzerland; 2 Dept. of Neurosurgery, Clinical Neuroscience Center, University Hospital Zurich and University of Zurich, Zürich, Switzerland; 3 Dept. of Neuroradiology, Clinical Neuroscience Center, University Hospital Zurich, Zürich, Switzerland; 4 cereneo Center for Neurology and Rehabilitation, Vitznau, Switzerland

**Keywords:** Crossed cerebellar diaschisis, ICA occlusion, BOLD MRI, Cerebrovascular reserve, Duplex sonography

## Abstract

Crossed cerebellar diaschisis (CCD) in internal carotid artery (ICA) stroke refers to attenuated blood flow and energy metabolism in the contralateral cerebellar hemisphere. CCD is associated with an interruption of cerebro-cerebellar tracts, but the precise mechanism is unknown. We hypothesized that in patients with ICA occlusions, CCD might indicate severe hemodynamic impairment in addition to tissue damage. Duplex sonography and clinical data from stroke patients with unilateral ICAO who underwent blood oxygen-level-dependent MRI cerebrovascular reserve (BOLD-CVR) assessment were analysed. The presence of CCD (either CCD+ or CCD−) was inferred from BOLD-CVR. We considered regions with negative BOLD-CVR signal as areas suffering from hemodynamic steal. Twenty-five patients were included (11 CCD+ and 14 CCD−). Stroke deficits on admission and at 3 months were more severe in the CCD+ group. While infarct volumes were similar, CCD+ patients had markedly larger BOLD steal volumes than CCD− patients (median [IQR] 122.2 [111] vs. 11.6 [50.6] ml; *p* < 0.001). Furthermore, duplex revealed higher peak-systolic flow velocities in the intracranial collateral pathways. Strikingly, posterior cerebral artery (PCA)-P2 velocities strongly correlated with the National Institute of Health Stroke Scale on admission and BOLD-CVR steal volume. In patients with strokes due to ICAO, the presence of CCD indicated hemodynamic impairment with larger BOLD-defined steal volume and higher flow in the ACA/PCA collateral system. Our data support the concept of a vascular component of CCD as an indicator of hemodynamic failure in patients with ICAO.

## Introduction

Crossed cerebellar diaschisis (CCD) refers to attenuated cerebral blood flow (CBF) and energy metabolism of the contralateral cerebellar hemisphere after supratentorial brain injury [1–3]. The term “diaschisis” was introduced by Monakow in 1914 (Greek *dia* “in half” and *schizien* “to split”), epitomizing a remote loss of excitability, induced by functional disconnection of brain regions (von Monakow 1914). Detection of CCD is achieved by the use of H_2_O position emission tomography, computed tomography perfusion imaging, and magnetic resonance imaging techniques, e.g. dynamic susceptibility contrast, arterial spin labelling, and most recently blood oxygenation-level-dependent (BOLD) functional MRI [4–7]. Although CCD was observed in non-vascular lesions such as glioma and does not require hemodynamic compromise [8], it has been best characterized in patients with middle cerebral artery (MCA) territory stroke [9, 10]. In patients with acute large vessel occlusion stroke affecting the MCA or internal carotid artery (ICA), the presence of CCD was shown to correlate with the size of the initial perfusion deficit [6]. However, its relationship to clinical outcome in stroke has been controversial [4, 6, 11]. We recently described CCD in patients at the sub-acute stage of symptomatic ICA occlusion (ICAO) using BOLD imaging of cerebrovascular reserve capacity (BOLD-CVR) [4]. ICAO may persist without clinical symptoms in patients with atherosclerotic disease, due to growth of compensatory vascular collaterals over time [12]. However, if hemodynamic impairment is not sufficiently compensated, recurrent clinical symptoms and strokes may occur. Duplex sonography parameters are useful to identify patients with ICAO whose collateral supply is insufficient and risk of recurrent ischemia higher [13, 14].

We hypothesized that in ICAO patients, in addition to stroke-induced tissue damage, hemodynamic impairment may contribute to CCD. Using duplex and BOLD-CVR, our goal was to describe a vascular component of CCD.

## Methods

### Study Design and Cohort Description

Patients with symptomatic ICA occlusions treated at the University Hospital Zurich Department of Neurology were prospectively collected from a BOLD-CVR database between October 2014 and October 2019. Some patient data of this cohort were previously reported [4, 5, 15]. Patients were excluded if they had a significant (medium- or high-grade) contralateral stenosis or occlusion of either the ICA or within the Circle of Willis. Patients were treated according to standard clinical practice. All patients gave written informed consent for data research and imaging procedures according to the study protocol. The study was approved by the local ethics committee (Kantonale Ethikkommission Zurich; KEK-ZH-Nr 2012-0427). In addition to routine head and neck vessel imaging and a complete duplex ultrasound of the brain-feeding arteries (extra- and intracranial, transforaminal, transorbital), patients received a BOLD MRI with a standardized carbon dioxide (CO_2_) challenge to measure CVR [4]. After initial hospitalization, patients had either a clinical follow-up after 3 months or a telephone interview to determine functional independence (modified Rankin Scale, mRS). The time interval between duplex and BOLD assessment was on average 28 days (median 5, min 0, max 279 days). Neurological deficits were graded by the treating stroke physicians using the National Institute of Health Stroke Scale (NIHSS). Clinical parameters, comorbidities, and risk factors were extracted from patients’ clinical charts.

### BOLD-CVR Imaging and Determination of CCD and Steal Phenomenon

The CO_2_ stimulus was administered using a computer-controlled gas blender with prospective gas targeting algorithms (RespirAct™, Thornhill Research Institute, Toronto, Canada). BOLD-CVR calculations using the combination of BOLD fMRI and the RespirAct™ have been shown to be highly reproducible [16]. The RespirAct™ allows for precise targeting of the arterial partial pressure of oxygen and CO_2_ [17]. During the BOLD-CVR study, all subjects were initially clamped at their own resting CO_2_ value [18]. CO_2_ was subsequently increased using a continuous step protocol of ~ 10 mmHg above their resting CO_2_ value for 80 s. Oxygen was maintained at a level of approximately 105 mmHg. All MRI data were acquired on a 3-T Skyra VD13 (Siemens Healthcare, Erlangen, Germany) with a 32-channel head coil. The BOLD fMRI parameters included an axial two-dimensional (2D) single-shot EPI sequence planned on the anterior commissure-posterior commissure line plus 20° (on a sagittal image), voxel size 3 × 3 × 3 mm^3^, acquisition matrix 64 × 64 × 35 ascending interleaved slice acquisition, slice gap 0.3 mm, GRAPPA factor 2 with 32 ref. lines, repetition time (TR)/echo time (TE) 2000/30 ms, flip angle 85°, bandwidth 2368 Hz/Px, and field of view 192 × 192 mm^2^. Two hundred volumes were acquired for the CVR study. A three-dimensional (3D) T1-weighted Magnetization Prepared Rapid Acquisition Gradient Echo image was also acquired with the same orientation as the fMRI scans for overlay purposes. The acquisition parameters included voxel size 0.8 × 0.8 × 1.0 mm^3^ with a field of view 230 × 230 × 176 mm and scan matrix 288 × 288 × 176, TR/TE/TI 2200/5.14/900 ms, and flip angle 8°. All BOLD fMRI volumes were pre-processed using SPM 12 (Statistical Parameter Mapping Software, Welcome Department of Imaging Neuroscience, University College of London, London, UK). Pre-processing included time and motion correction and smoothing with a Gaussian kernel of 6 mm full-width at half-maximum. The T1 image was aligned to the mean BOLD volume to allow the T1 to function as an anatomical overlay. The T1 was then segmented into grey matter, white matter, cerebrospinal fluid, skin, and skull probability maps. Only voxels with a combined probability of > 0.8 for grey or white matter were considered for further analysis. Construction of final BOLD-CVR maps was done using in-house MATLAB R2019a scripts. To correct for difference in delay between the CO_2_ time series and BOLD fMRI time series on a voxel-by-voxel basis, a temporal smoothing of the BOLD fMRI signal time courses with a high-pass filter was done. Afterwards, the CO_2_ time series was corrected on a voxel-wise basis using a previously written and published algorithm [19], so that the increase in CO_2_ corresponds in time with the response of the BOLD fMRI. After this voxel-wise time correction, BOLD-CVR, defined as the % BOLD fMRI signal change/mmHg CO_2_, was calculated from the slope of a linear least square fit of the BOLD signal time course to the CO_2_ time series over the whole time series [19]. The application in stroke patients has also been described previously [4, 5]. For each patient, individual hemispheric cerebellar masks were created with a subjects’ specific subcortical anatomic allotment using Freesurfer software (http://surfer.nmr.mgh.harvard.edu) [20, 21]. These cerebellar masks were then used as a region of interest to determine BOLD-CVR within the whole ipsilateral cerebellar hemisphere and the whole contralateral—*crossed—*cerebellar hemisphere. This allowed determining the BOLD-CVR cerebellar asymmetry index (i.e. the percentage difference between both cerebellar hemispheres). Classification into CCD positive (CCD+) or CCD negative (CCD−) was then achieved using a BOLD-CVR cerebellar asymmetry index of > 6%, as previously described [4]. Brain areas exhibiting steal phenomenon ipsilateral to the ICAO were determined based on negative BOLD-CVR. All voxels surrounding the anterior cranial fossa and within ventricles were excluded to reduce the number of artificially negative BOLD-CVR values. All voxels showing negative BOLD were taken and the volume of negative BOLD-CVR values was then identified by determining the volume of negative BOLD-CVR voxels on the T1-weighted image (i.e. (voxel size: 0.8 × 0.8 × 1) × 1000 = ml).

### Duplex Imaging

Colour-coded duplex ultrasound imaging of the extra- and intracranial (transtemporal/transorbital/transforaminal) arteries was performed on a Siemens Accuson X 2000 clinical duplex scanner according to standardized protocols within the Department of Neurology clinical routine. Vascular segments were described previously [13]. We recorded peak-systolic velocities (PSV) and end-diastolic velocities (EDV) in centimetres per second. To classify routes of collateral flow, the four main collateral pathways were identified according to [14, 22] as (1) anterior communicating artery (ACoaA), (2) posterior communicating artery (PCoA), (3) leptomeningeal, and (4) ophthalmic artery (OA) collaterals.

### Analysis of Infarct Volume

Infarct volume was determined from diffusion-weighted MRI images (DWI) acquired in clinical routine on average 5 days (median 2, min 0, max 23) after stroke using the software ImageJ (NIH, version 2.0.0/rc/67/1.52 s). We outlined the hyperintense lesion slice by slice with a semi-automated thresholding tool and calculated infarct volume by multiplying pixel number with pixel size and slice thickness (+ interslice gap).

### Statistical Analyses

Statistical analyses were performed using SPSS (IBM, Vs 25). For group comparisons, we used the non-parametric Fisher’s exact test (categorical measurements) and 2-tailed Mann-Whitney *U* test (continuous measurements). For bivariate correlation analyses of scales, the Pearson correlation coefficient was calculated. *p* values < 0.05 were considered significant. For correlations with ordinal values like stroke severity scores, the Spearman correlation coefficient was used.

## Results

**Fig. 1:**
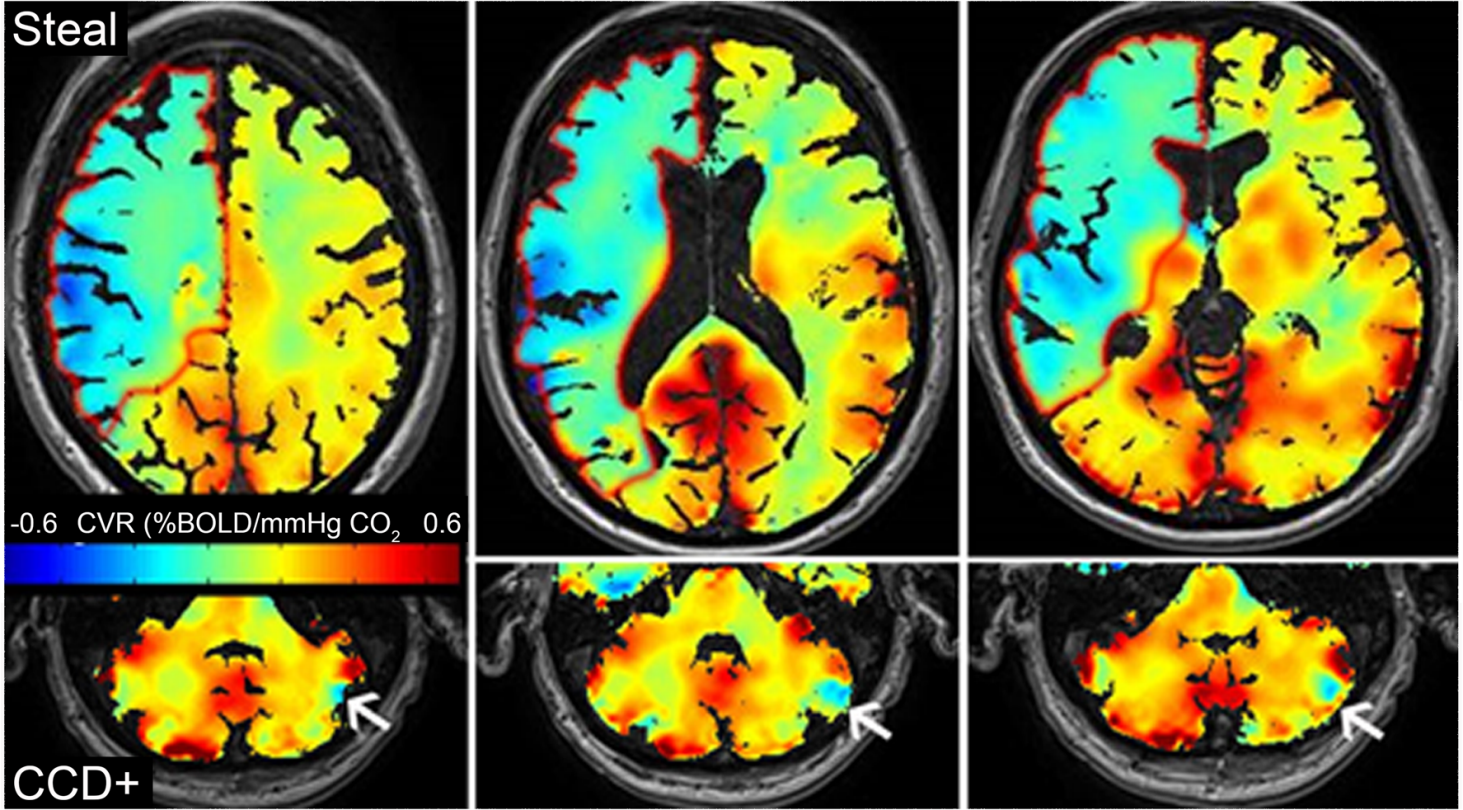
BOLD-CVR assessment of CCD. Example of a patient with hemodynamic impairment in the right MCA territory due to right ICA occlusion. Three slices are shown. Steal phenomenon (i.e. negative BOLD-CVR) is depicted in green-blue and outlined in red on the upper panel. The lower panel shows three cerebellar slices of the same patient. Reduced CVR is observed on the contralateral cerebellar hemisphere (white arrow)

Of 39 patients with symptomatic ICAO initially identified in the database, seven were excluded because of contralateral ICA stenosis or additional intracranial stenosis within the Circle of Willis. Furthermore, three patients were excluded due to insufficient or missing imaging or duplex data, and three due to uncertain symptom onset. Of the remaining 25 patients, 11 were classified CCD+ and 14 CCD−. A typical BOLD-CVR image of a CCD+ patient is shown in Fig. 1.

Clinical characteristics of CCD+ and CCD− groups are summarized in Table 1. Furthermore, characteristic MRI (DWI, FLAIR, BOLD-CVR) and direct stroke corticospinal tract involvement (CST) for each patient are shown in Table 2. Sex, age, risk factors, and stroke etiologies were similar in both groups. Patients in the CCD+ group had a significantly higher NIHSS and mRS on admission (CCD+ vs. CCD−: median [IQR]: NIHSS 6 [8] vs. 0 [3], *p* = 0.005; mRS 3 [2] vs. 0 [3], *p* = 0.031). Neurological deficit at 3 months was also more severe in the CCD+ group (NIHSS 3 months CCD+ vs. CCD−: median [IQR] 1 [1] vs. 0 [2], *p* = 0.023), while the difference in mRS at 3 months was not significant (CCD+ vs. CCD−: median [IQR] 1 [1] vs. 0 [1], *p* = 0.059). Timing of BOLD-CVR imaging in relation to clinical symptoms of ischemia was variable, since this technique is only used in selected stroke patients in our center. However, the distance between symptoms and BOLD was not different in CCD+ or CCD− patients (median [IQR] 7 [61] vs. 5 [23.75] days, *p* = 0.803). Infarcts involving the corticospinal tract (either on DWI or on FLAIR images, see Table 2) were not more frequent in patients with CCD (*p* = 0.265).

**Table 1:** Patient clinical characteristics

	All *n* = 25 (%)	CCD+ *n* = 11 (%)	CCD− *n* = 14 (%)	*p* value
Demographic data				
Age (range)	65 (33–81)	66 (49–77)	64 (33–81)	0.979
Female	6 (24)	3 (27.3)	3 (21.4)	1
Medical history, *n* (%)				
Smoking	15 (60)	9 (81.8)	6 (42.9)	0.099
Hypertension	20 (80)	10 (90.9)	10 (71.4)	0.341
Diabetes	5 (20)	3 (27.3)	2 (14.3)	0.623
CAD	8 (32)	3 (27.3)	5 (35.7)	0.695
pAOD	4 (16)	2 (18.2)	2 (14.3)	1
Dyslipidemia	17 (68)	7 (63.6)	10 (71.4)	1
Atrial fibrillation	3 (12)	2 (18.2)	1 (7.1)	0.565
Clinical scores, median (IQR)				
NIHSS on admission	3 (5)	6 (8)	0 (3)	0.005*
NIHSS at 3 months	0 (3)	1 (1)	0 (2)	0.023*
mRS on admission	2 (3)	3 (2)	0 (3)	0.031*
mRS at 3 months	1 (1)	1 (1)	0 (1)	0.059*
TOAST, subtype, *n* (%)				
Large vessel disease	15 (60)	6 (54.5)	9 (64.2)	0.856
Cardio embolic	0 (0)	0 (0)	0 (0)	
Small artery disease	0 (0)	0 (0)	0 (0)	
Other causes	5 (20)	3 (27.3)	2 (14.3)	
Undetermined	5 (20)	2 (18.2)	3 (21.4)	

Clinical characteristics of all 25 - patients (all) and patient groups without (CCD−) or with (CCD+) crossed cerebellar diaschisis. *p* values < 0.05 in Mann-Whitney *U* test or Fisher exact test are marked with asterisk. CAD coronary artery disease, *pAOD* peripheral artery occlusive disease. Numbers (*n*) and percentage or median and interquartile range (IQR) are shown

**Table 2:**
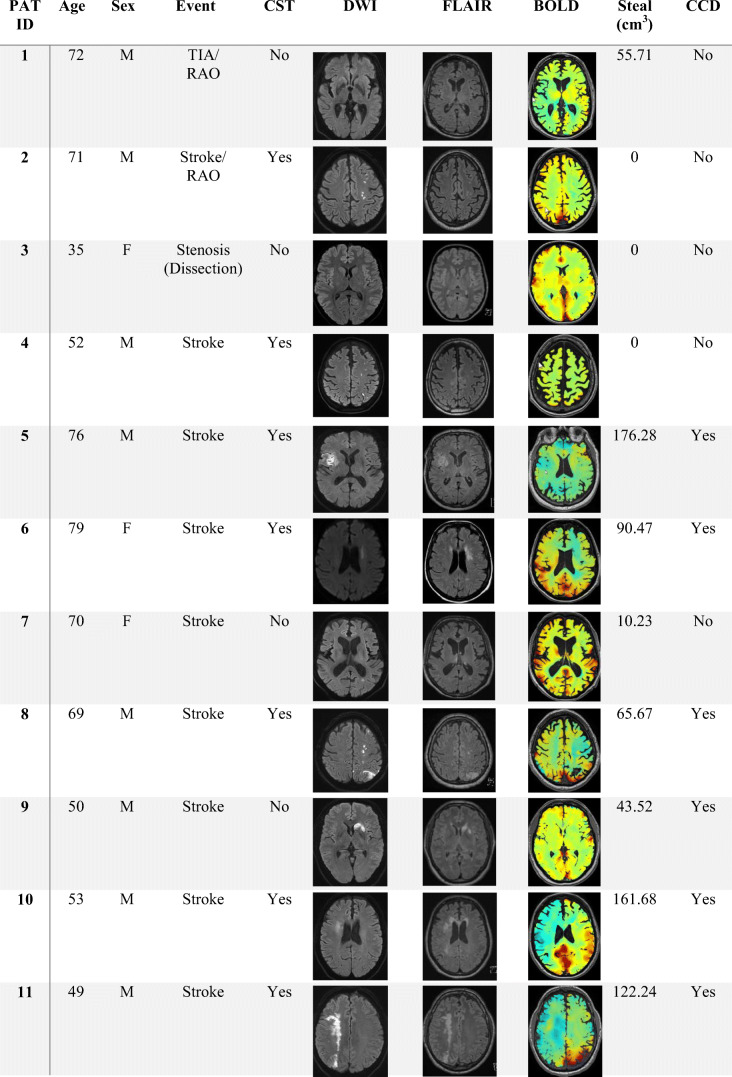

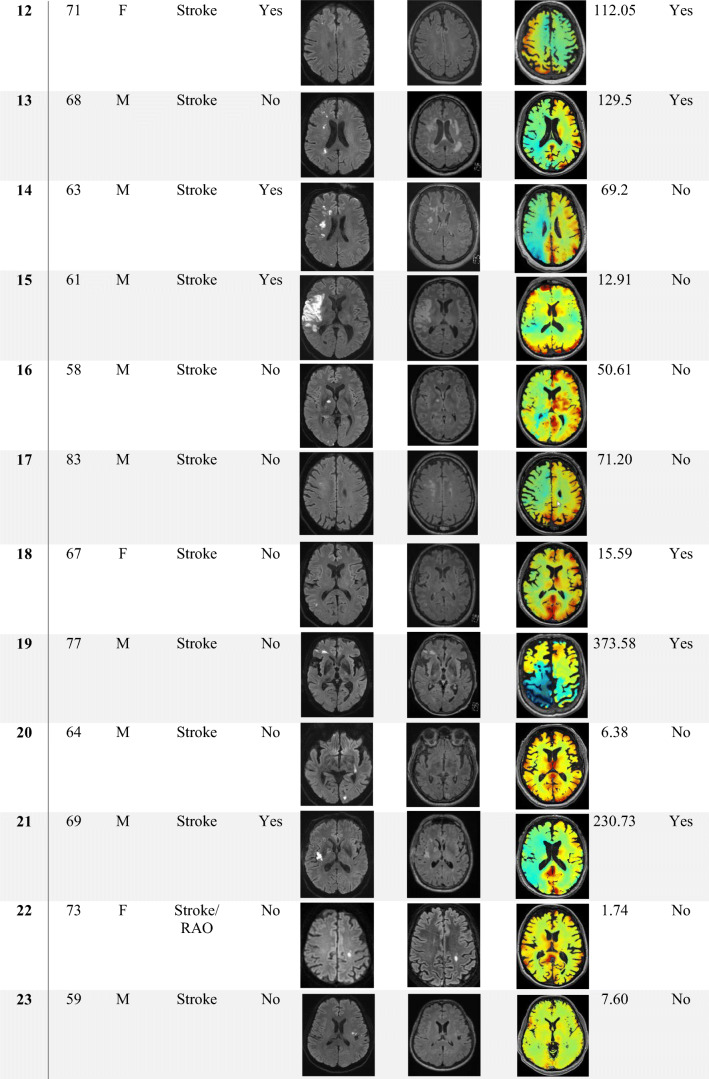

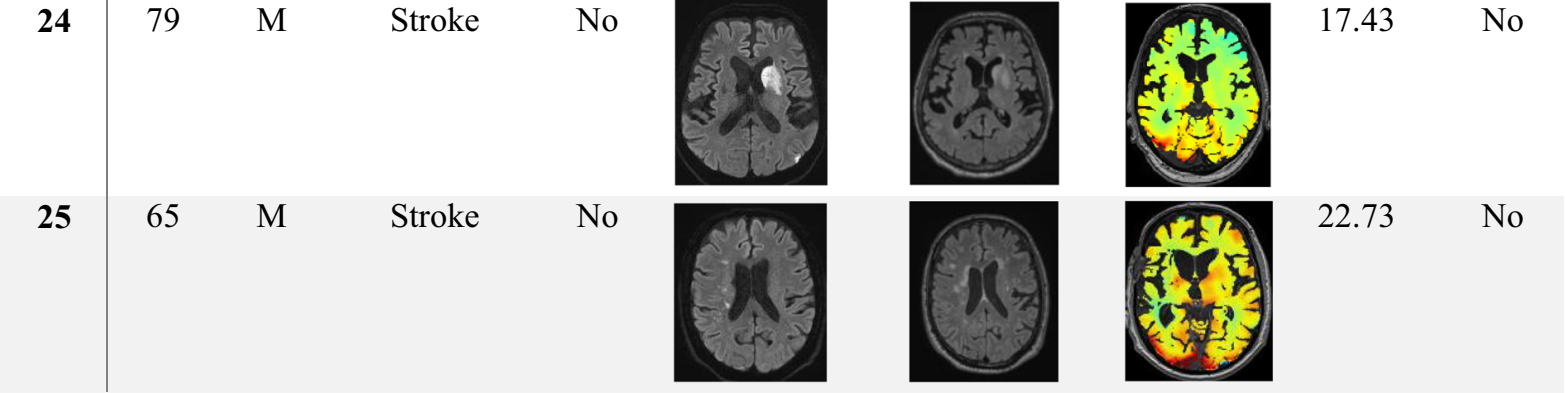
Patient imaging findings

Duplex and BOLD parameters, displayed in Table 3, demonstrate that there were increased flow velocities in CCD+ patients in the basilar artery (BA), contralateral PCA P1 and P2 segments, ipsilateral P2 segment, and anterior cerebral artery (ACA). Median CCD+ flow velocities in the ipsilateral PCA P1 segment were higher as well, but this trend did not reach significance. MRI determined infarct volumes were small and similar in both patients’ groups. As expected, supratentorial BOLD-CVR was lower at the side of ICA occlusion compared with the contralateral side in all included patients. However, although the magnitude of hemispheric BOLD-CVR reduction was similar in CCD+ and CCD− patients, the most striking finding from BOLD imaging data was a markedly larger steal volume on the ipsilateral supratentorial hemisphere in CCD+ patients (median [IQR]: CCD+ 122.2 [111] vs. CCD− 11.6 [50.6] ml; *p* < 0.001).

**Table 3:** Duplex and BOLD parameters

Duplex parameter (in cm/s)	All *N* = 25	CCD+ *N* = 11	CCD− *N* = 14	*p* value
Intracranial				
PSV ACA ipsi	67 (45; 25)	62 (40; 11)	79 (57; 14)	0.819
EDV ACA ips	32 (32; 25)	26 (11; 11)	34 (41; 14)	0.757
PSV ACA contra	108 (86; 23)	121 (102; 9)	97 (70; 14)	0.128
EDV ACA contra	47 (45; 23)	59 (49; 9)	40.5 (38; 14)	0.015*
PSV M1 ipsi	79.5 (52; 24)	73 (44; 11)	88 (50; 13)	0.234
EDV M1 ipsi	37 (21; 24)	32 (12; 11)	41 (35; 13)	0485
PSV M1contra	115 (37; 25)	119 (49; 11)	114.5 (38; 14)	0.600
EDV M1 contra	45 (20; 25)	47 (20; 11)	40.5 (20; 14)	0.161
PSV P1 ipsi	74 (52; 25)	84 (44; 11)	73 (72; 14)	0.676
EDV P1 ipsi	30 (23; 25)	34 (21; 11)	28.5 (29; 14)	0.491
PSV P1 contra	69.5 (31; 24)	81.5 (40; 10)	56 (32; 14)	0.003*
EDV P1 contra	27 (14; 24)	30 (13; 10)	20.5 (16; 14)	0.027*
PSV P2 ipsi	66 (29; 25)	76 (33; 11)	51 (15; 14)	< 0.001*
EDV P2 ipsi	27 (13; 25)	37 (17; 11)	24 (8; 14)	0.006*
PSV P2 contra	59 (33; 25)	74 (22; 11)	46 (19; 14)	0.001*
EDV P2 contra	20 (15; 25)	24 (19; 11)	18 (9; 14)	0.027*
Transforaminal				
PSV BA	66 (38; 25)	79 (39; 11)	55.5 (25; 14)	0.043*
EDV BA	26 (11; 25)	26 (19; 11)	25.5 (11; 14)	0.474
MRI parameter				
Infarct volume (cm^3^)	3 (8.6; 25)	3.7 (11.9; 11)	1.3 (8.5; 14)	0.105
CST involvement (*n*, %)	13 (52)	7 (63.6)	6 (42.9)	0.265
CVR supra ipsi	0.08 (0.11; 25)	0.04 (0.09; 11)	0.09 (0.06; 14)	0.095
CVR supra contra	0.14 (0.08; 25)	0.14 (0.09; 11)	0.16 (0.09; 14)	0.609
CVR cerebellar ipsi	0.17 (0.09; 25)	0.18 (0.17; 11)	0.16 (0.07; 14)	0.472
CVR cerebellar contra	0.16 (0.08; 25)	0.14 (0.18; 11)	0.16 (0.06; 14)	0.785
Steal volume (cm^3^)	50.6 (108; 25)	122.2 (111; 11)	11.6 (50.6; 14)	<0.001*

Duplex assessment of flow (PSV/EDV) or BOLD MRI assessment (for CVR) measured ipsi—or contralateral to ICAO (“ipsi” vs. “contra”) in all patients (all) and patients’ groups with or without CCD. *p* values < 0.05 in Mann-Whitney *U* test are marked with asterisk. Median (IQR; *n*) is shown

**Fig. 2:**
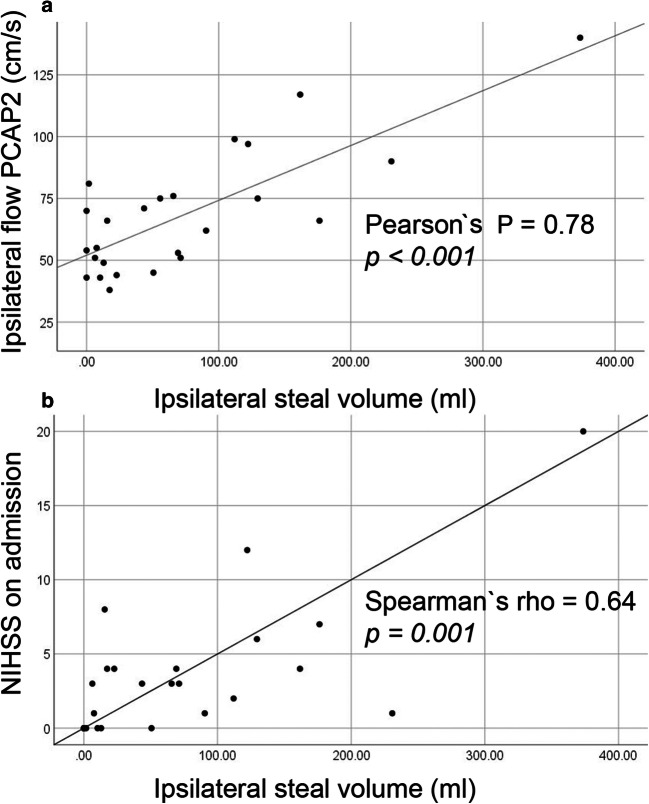
Correlation of hemodynamic and clinical parameters in ICA occlusion patients. Scatter plots of bivariate correlation analyses between ipsilateral BOLD-CVR-steal volume (ml) on the *x*-axis, and **a** flow in the P2 segment of the ipsilateral PCA (cm/s) as well as **b** NIHSS on admission. Results of bivariate correlation analyses from all patients show a significant linear correlation

In a next step, we searched for a correlation between individual duplex and BOLD-CVR parameters. We found a strong linear correlation between volume of steal phenomenon and flow velocity in the P2 segment of both PCAs (Pearson’s *P* for ipsilateral PCA-P2 0.787, *p* < 0.001, for contralateral PCA-P2 0.753, *p* < 0.001; shown for the ipsilateral P2 in Fig. 2a), as well as between steal volume and severity of initial neurological deficits on admission NIHSS (Spearman’s rho 0.64, *p* < 0.001: Fig. 2b).

## Discussion

In its original description, CCD was regarded a neurophysiological phenomenon, caused by disconnection of cortico-ponto-cerebellar tracts with deactivation of cerebellar Purkinje cells [23–25]. Gold and Lauritzen were able to elicit this neuronal deactivation by experimental ischemia as well as functional ablation of the cerebral cortex in rats, and provided the direct link to concordant decreases in cerebellar blood flow along with the depression in neural activity [26].

However, for a long time, the relevance of CCD for clinical practice was uncertain, because no clear clinical correlate of disconnection was found in stroke patients. Recently, a wider definition of diaschisis was suggested, accounting for different aspects of remote brain dysfunction after a lesion: focal versus non-focal (connectional or connectomal) [27]. CCD as it occurs in supratentorial stroke would be a focal type of diaschisis, but it is likely that other types of network alterations occur in focal stroke as well.

Previous work suggested that patients with MCA territory stroke and CCD had more severe supratentorial hypoperfusion and hemodynamic impairment [4, 6, 7, 10]. We hypothesized that in these patients, CCD might have a potential vascular component, incorporating compensatory blood flow responses to persisting vascular occlusions affecting the MCA territory.

**Fig. 3:**
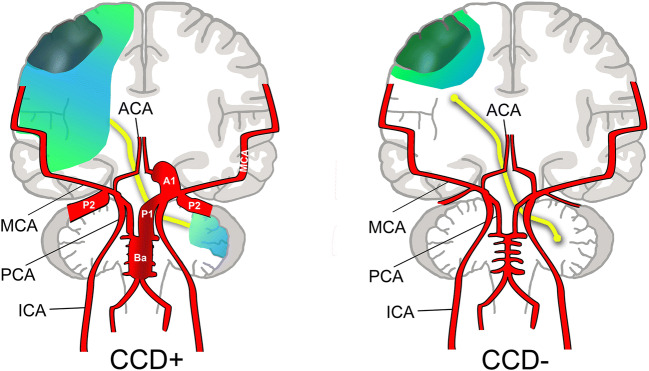
Concept for a vascular component in CCD. In CCD+ patients, even with a small stroke that may have a similar volume to CCD− patients (dark green area), hemodynamic impairment results in a large BOLD-defined ipsilateral steal volume (light blue/green area). In order to compensate hemodynamic impairment in CCD+ patients, collateral efforts are strongly increased (broadened vessel diameters in CCD+). This manifests as increased flow within the contralateral ACA (A1), supplying the retrograde ipsilateral ACA, as well as the vertebrobasilar system (BA, P1, P2)

In this study, we demonstrate that indeed the brain area affected by severe hemodynamic compromise (i.e. steal phenomenon) was by far larger in CCD+ patients (median BOLD-CVR steal volume of approximately 122 vs. 12 ml). This went along with a more severe neurological deficit on admission (median NIHSS 6 vs. 0) and at 3 months (median NIHSS 1 vs. 0). Furthermore, we were able to reveal increased hemodynamic efforts in CCD+ patients by routine duplex: flow in collateral pathways was higher, particularly in those collateral routes coming from the posterior circulation (BA/PCA). This is similar to our findings in patients with ICA occlusion that were at higher risk of recurrent ischemia [13], where flow in bilateral PCA-P2 segments was higher. In our cohort, flow in the PCA on both sides correlated to supratentorial steal volume on the side of ICAO. Supratentorial steal volume also correlated with initial neurological deficit on admission (NIHSS). Based on our findings, we suggest that in patients with ICA occlusion, CCD+ shows a propensity towards more hemodynamic compromise and compensatory collateral flow via the Circle of Willis (CoW; Fig. 3). It is unlikely that these PCA flow increases cause the observed decrease in blood flow or BOLD signal within the contralateral cerebellar hemisphere in CCD+. This was confirmed by our BOLD data, revealing no overall differences in hemispheric cerebellar BOLD-CVR, except for the cerebellar asymmetry index.

One limitation of our study is the small sample size (*n* = 25). Furthermore, the time interval between stroke and BOLD imaging, albeit not different between CCD+/CCD− groups, was highly variable. Of note, patients in this study were not consecutively enrolled stroke patients with ICA occlusion, but selected patients without M1 or other intracranial artery occlusions that were able and willing to participate in additional BOLD-CVR imaging. Therefore, there was a selection bias towards less severely affected patients. Another limitation is that information about initial perfusion/mismatch status was missing for many patients, so that hemodynamic characterization of the hyper-acute stroke phase was not possible. However, while different hemodynamic factors such as the extent of collaterals may shape the severity of the initial stroke, our findings indicate that hemodynamic impairment persists beyond the hyper-acute phase of stroke. Infarct size is not an indicator of hemodynamic compromise. In contrast, BOLD-CVR imaging and duplex can help to identify patients with CCD and lingering deficits in cerebrovascular reserve.

## Summary

We suggest that ipsilateral supratentorial hemodynamic impairment due to large vessel occlusion in CCD+ patients elicits complex neurovascular reactions leading to distant CBF changes. Therefore, despite a similar amount of supratentorial stroke-induced tissue damage, CCD+ patients with ICAO had more severe hemodynamic impairment, reflected by larger supratentorial steal volumes in BOLD-CVR and increased compensatory collateral flow in the posterior circulation. This suggests a vascular component of CCD. Furthermore, CCD+ patients had more severe clinical symptoms on admission and after 3 months, indicating that severe hemodynamic impairment may induce more severe clinical deficits leading to less favourable functional recovery. Radiological infarct size estimates may therefore not capture the hemodynamic source of clinical deficits (clinical/radiological mismatch). Our findings support the use of non-invasive hemodynamic and cerebral blood flow measurements to characterize stroke patients with persisting vascular occlusion to detect impaired hemodynamic capacity. Future studies with serial BOLD-CVR measurements in patients with vascular disease may reveal if CCD changes over time in a way that is related to the clinical course.
